# Human Adenovirus Entry and Early Events during Infection of Primary Murine Neurons: Immunofluorescence Studies In Vitro

**DOI:** 10.3390/pathogens13020158

**Published:** 2024-02-09

**Authors:** Anna Słońska, Aleksandra Miedzińska, Marcin Chodkowski, Piotr Bąska, Aleksandra Mielnikow, Michalina Bartak, Marcin W. Bańbura, Joanna Cymerys

**Affiliations:** 1 Division of Microbiology, Department of Preclinical Sciences, Institute of Veterinary Medicine, Warsaw University of Life Sciences, 02-786 Warsaw, Poland; ol.miedzinska@gmail.com (A.M.); mielnikow.aleksandra@gmail.com (A.M.); michalina_bartak@sggw.edu.pl (M.B.); marcin_banbura@sggw.edu.pl (M.W.B.); joanna_cymerys@sggw.edu.pl (J.C.); 2 Military Institute of Hygiene and Epidemiology, Kozielska 4, 01-163 Warsaw, Poland; marcin.chodkowski@wihe.pl; 3 Division of Pharmacology and Toxicology, Department of Preclinical Sciences, Institute of Veterinary Medicine, Warsaw University of Life Sciences-SGGW, 02-786 Warsaw, Poland; piotr_baska@sggw.edu.pl

**Keywords:** human adenovirus (HAdV), primary murine neurons, entry, endocytosis, early endosomes

## Abstract

Human adenovirus (HAdV) is a common pathogen, which can lead to various clinical symptoms and—in some cases—central nervous system (CNS) dysfunctions, such as encephalitis and meningitis. Although the initial events of virus entry have already been identified in various cell types, the mechanism of neuronal uptake of adenoviruses is relatively little understood. The aim of this study was to investigate early events during adenoviral infection, in particular to determine the connection between cellular coxsackievirus and adenovirus receptor (CAR), clathrin, caveolin, and early endosomal proteins (EEA1 and Rab5) with the entry of HAdVs into primary murine neurons in vitro. An immunofluorescence assay and confocal microscopy analysis were carried out to determine HAdV4, 5, and 7 correlation with CAR, clathrin, caveolin, and early endosomal proteins in neurons. The quantification of Pearson’s coefficient between CAR and HAdVs indicated that the HAdV4 and HAdV5 types correlated with CAR and that the correlation was more substantial for HAdV5. Inhibition of clathrin-mediated endocytosis using chlorpromazine limited the infection with HAdV, whereas inhibition of caveolin-mediated endocytosis did not affect virus entry. Thus, the entry of tested HAdV types into neurons was most likely associated with clathrin but not caveolin. It was also demonstrated that HAdVs correlate with the Rab proteins (EEA1, Rab5) present in early vesicles, and the observed differences in the manner of correlation depended on the serotype of the virus. With our research, we strove to expand knowledge regarding the mechanism of HAdV entry into neurons, which may be beneficial for developing potential therapeutics in the future.

## 1. Introduction

Human adenoviruses (HAdVs) are widespread infectious agents, which can lead to various clinical symptoms, including upper and lower respiratory tract infections, gastroenteritis, hepatitis, or keratoconjunctivitis [1,2]. The manifestations of HAdV infections may also include central nervous system (CNS) dysfunctions, such as encephalitis and meningitis, especially in immunocompromised patients and children. CNS dysfunction associated with adenovirus infection typically occurs in the setting of pulmonary or disseminated disease in other body sites [3,4]. Although the reports on this issue are limited to a few case reports, certain serotypes of adenoviruses—including type 1, 2, 3, 5, or 7—have been isolated from brain tissue and cerebrospinal fluids (CSF) [3,4,5,6].

Adenovirus entry into the cell is a complex process, which requires a series of cellular factors [7,8]. The attachment of most HAdV types is mediated by the coxsackievirus and adenovirus receptor (CAR), which is expressed in numerous mammalian tissues, including the brain, heart, or lung [9]. CAR-docked particles activate integrin receptors, triggering endocytosis [10,11]. The main endocytic pathways used by adenoviruses to enter host cells are clathrin-dependent endocytosis (HAdV2, HAdV5) and macropinocytosis (HAdV3) [10,12]. In clathrin-dependent endocytosis, there is a significant interaction between the protein fibers of adenovirus capsids and the CAR cell surface receptor. Signals from integrins (αvβ3 and αvβ5)—thanks to CAR receptors and formation of the ligand–receptor complex—lead to the activation of p130cas protein, Rab5, PI3 kinase, proteins belonging to the Rho/GTPase family (Cdc42, Rac), and rearrangement of the actin cytoskeleton [8,9]. In the next stage of infection, the virus is enclosed in clathrin-coated vesicles (CCV) [13,14,15]. In turn, HAdV3 uses macropinocytosis as its endocytic pathway in epithelial and hematopoietic cells. Contrary to the previously mentioned HAdV2 and HAdV5, it does not use the CAR receptor but the CD46 receptor [7,8]. As a result of interaction of the virus with the CD46 receptor, Rab5 and PI3 kinase are activated, and the viral protein/integrin complex is formed [12,16,17]. The Cdc42 and Rac1 proteins contribute to the polymerization of actin and formation of the macropinosome.

Small Rab GTPases play a regulatory role in the transformations of cell membranes and are involved in coordinating viral endocytosis. One of the major members of the Rab family are Rab5 proteins, which are located in the membrane of clathrin-coated vesicles and the membrane of early endosomes (EE) [18]. Rab5 proteins are critical regulators of the early stages of endocytosis and early endosome fusion in vitro. In the membranes of early endosomes, these proteins appear in the form of clusters. The essence of Rab proteins’ activity is their interaction with effector proteins, including the early endosome antigen 1 (EEA1) protein—the regulator of early endosome fusion [19]. EEA1 is implicated in docking the incoming endocytic vesicles before fusion with early endosomes. 

The mechanism of HAdV entry into neurons is not well understood. Although the incidence rate of CNS dysfunction associated with adenovirus infection is relatively low, it can still be a severe complication, especially in children who develop an adenovirus infection. Therefore, the research presented aims to complement and expand the knowledge regarding early events during HAdV entry into neurons. Considering that clathrin-mediated endocytosis has become known as the major uptake pathway used by HAdV [4], we investigated its role during HAdV entry. To better understand the mechanism of neuronal uptake of adenoviruses, we attempted to determine the correlations be-tween CAR, clathrin, caveolin, early endosomes, and HAdV and to investigate the effect of selected drugs causing inhibition of clathrin- and caveolin-dependent endocytosis on the efficiency of HAdV infection in primary murine neurons.

## 2. Materials and Methods

### 2.1. Cells and Adenoviruses

The primary culture of murine neurons was prepared and cultured as described previously [20]. Neurons were suspended in B-27 Neuron Plating Medium consisting of neurobasal medium, B27 supplement, glutamine (200 mM), glutamate (10 mM), antibiotics (penicillin and streptomycin) with 10% supplement of fetal and equine serum (Thermo Fisher Scientific, Waltham, MA, USA). Neurons were infected with three human adenovirus types: HAdV4 (species E, RI-67, ATCC VR-1572), HAdV 5 (species C, Adenoid 75, ATCC VR-5), or HAdV 7 (species B, Gomen, ATCC VR-7) 10^5^ CCID_50_/mL in culture medium (MOI = 0.2 PFU/cell).

### 2.2. Infection of Cultured Murine Neurons with HAdVs

Primary murine neurons designated for the immunofluorescence assay were plated onto poly-D-lysine and laminin-coated sterile coverslips placed in 6-well culture plates (1 × 10^5^ cells per well). Cells were infected with HAdV4, 5, or 7, and after a 60 min incubation at 37 °C, the viral inoculum was removed, and fresh growth medium was added. Next, the infected neurons were incubated for 15, 30, 60 min, and 2 h at 37 °C with 5% CO_2_. Control, mock-infected cells were processed under the same conditions, but they were not infected with HAdVs.

### 2.3. Immunofluorescence Assay

For immunofluorescence staining, after 15, 30, 60, 120 min pi, cells were fixed in 3.7% paraformaldehyde/PBS (Sigma-Aldrich, St. Louis, MO, USA), for 30 min at room temperature, permeabilized in 0.5% Tween/PBS for 5 min, washed twice in PBS, and blocked with PBS containing 1% bovine serum albumin (BSA) (Sigma-Aldrich, St. Louis, MO, USA). For evaluation of the role of EEA1 and Rab5, anti-EEA1 monoclonal antibody (dilution 1:200; MA5-14794) and anti-Rab5 polyclonal antibody (dilution 1:100; PA5-29022) (Thermo Fisher Scientific, Waltham, MA, USA) were added for 60 min at 37 °C. Subsequently, unbound antibodies were removed by washing in PBS, and then, Texas Red-X goat anti-rabbit IgG (dilution 1:1000; Thermo Fisher Scientific, Waltham, MA, USA) was added for 60 min. Anti-CAR rabbit polyclonal IgG (dilution 1:100, overnight at 4 °C; BS-2389R) and the corresponding secondary antibody Texas Red-X goat anti-rabbit IgG (Thermo Fisher Scientific, Waltham, MA, USA) were used for receptor detection. Caveolin and clathrin were also stained, employing indirect immunofluorescence with monoclonal antibodies: caveolin-1 mouse monoclonal IgG (SC-53564) and clathrin goat polyclonal IgG (SC-6579) (Santa Cruz Biotechnology, Dallas, TX, USA) (dilution 1:100, 1 h, at 37 °C). To visualize the reaction, secondary antibodies Alexa Fluor 660 donkey anti-goat IgG and Alexa Fluor 569 goat anti-mouse IgG (Invitrogen) were used (dilution 1:250, 1 h, at RT in darkness). The presence of viral antigen was detected through direct immunofluorescence using mouse Adenovirus FITC-labeled monoclonal antibody (Cat. No. 5016, Light Diagnostics™, Merck Millipore, Darmstadt, Germany). Cell nuclei were stained with Hoechst 33,258 in compliance with the manufacturer’s recommendations.

### 2.4. Image Acquisition and Confocal Microscopy

Stained coverslips were mounted on glass slides in ProLong Gold Antifade Reagent and examined using Fluoview FV10i laser scanning confocal microscope (Olympus Poland Sp. z o.o.) with 60× water-immersion objective, using ultraviolet/visible light LD lasers. Images were captured using FV10i software (resolution 1024 × 1024) and converted to 24-bit tiff files for visualization. Images were collected using z-stack imaging and analyzed using FV10i software (Olympus), the Fiji version of the free image processing software ImageJ (NIH Image, version 1.53a, Bethesda, MD, USA), and Adobe Photoshop CS6 software (Adobe Systems Incorporated, ver. 13.0, San Jose, CA, USA).

### 2.5. Image Processing and Correlation Analysis

The correlation analysis of HAdV antigen with CAR, caveolin, clathrin, EEA1, or Rab5 was performed by employing the Coloc 2 plugin of the extended ImageJ version Fiji. The micrographs in all figures were performed on maximum intensity projections to select pixels of the highest intensity from every slice throughout the volume to construct a 2D image. Based on pixel-intensity correlation measurements, Pearson’s coefficient correlation (r) was evaluated according to the protocol [https://imagej.net/plugins/coloc-2, accessed on 9 February 2023]. After importing confocal images into the Fiji software, the color channels were split into separate images, and the region of interest (ROI) for performing correlation analysis was selected. Pearson’s coefficient correlations were calculated from ten independent fields of cells in two different experiments, and the values were presented as mean ± SD. The degrees of correlation were indicated as perfect for values 0.09–1.00, strong for values between 0.70 and 0.89, moderate for values between 0.40 and 0.69, weak for values 0.10–0.39, and negligible for values 0.00–0.10 [21,22].

For each indicated HAdV-infected cell, the fluorescence intensity measurement was performed. The signals corresponding to red (CAR/clathrin/caveolin/EEA1/Rab5) and green (HAdV antigens) channels were measured along the yellow line and presented as line profile plots. Intensity plots were also performed on maximum intensity projections.

### 2.6. Endocytosis Inhibitors

Chlorpromazine and nystatin were purchased from Sigma-Aldrich. A stock solution of chlorpromazine was obtained in double deionized water, whereas nystatin was dissolved in dimethylsulfoxide (DMSO). The final concentration of DMSO was less than 0.5%. Aliquots of the stock solutions were stored at −20 °C until use.

### 2.7. Cell Viability Assay

To find an optimal concentration of inhibitors, which did not affect primary neurons’ viability, the XTT assay (Sigma-Aldrich, St. Louis, MO, USA), was performed according to the manufacturer’s protocol. Briefly, neurons were cultured on 96-well microplates in the medium containing different concentrations of each inhibitor. Chlorpromazine treatments of 1.25, 2.5, 5, 10, 25, 50 µM or nystatin treatments of 6.25, 12.5, 25, 50, 75, 100 µM were administered in 100 µL of media/well, and cells were incubated for 3 and 24 h at 37 °C. At the end of exposure, 50 μL of the activated XTT solution was added to each well and incubated for 4 h at 37 °C and 5% CO_2_. Measurement of the absorbance of the blank background control and samples was performed using a microplate reader (Synergy H1, BioTek Instruments, Inc., Winooski, VT, USA) at a wavelength between 450 and 500 nm. The reference wavelength was between 650 and 690 nm. The obtained results were normalized to the control samples, where cell viability was set to 100%. Cell viability was expressed as a percentage of viability of the control culture. All experiments were performed in triplicate.

### 2.8. Inhibition of HAdV Replication by Endocytosis Inhibitors

Neurons were seeded in 6-well plates and cultured as described before. Next, the media were removed, and cells were pre-treated with increasing amounts of the inhibitors (chlorpromazine: 2.5, 5, 10 µM; nystatin: 12.5, 25, 50 µM) 1 h prior to infection. Subsequently, the media were removed, HAdV stock (CCID_50_ = 10^5^/mL, MOI = 0.2) was overlaid on the cells in the presence of inhibitors, and cultures were incubated at 37 °C for 1 h. Unbound virus particles were removed by rinsing the cells in PBS. The infected neurons were further cultured in fresh culture medium supplemented with endocytosis inhibitors at 37 °C. At 2 h pi, viral DNA was isolated, and its yield was quantified with quantitative real-time PCR.

### 2.9. Quantitative Real-Time PCR

A 841 bp long fragment of Ad5 was amplified using primers Adv_full_L (GTAAGCTTGATCCCCGCCCT) and Adv_full_R (GCCGCGGATGTCAAAGTACG). The product was resolved in 1% agarose gel, followed by band excision and purification using GeneJET Gel Extraction Kit (Thermo Fisher Scientific, Waltham, MA, USA). The product was ligated with DNA of pGEM*^®^*-T vector (Promega, Madison, WI, USA) and cloned in E. coli JM109 (Promega, Madison, WI, USA), resulting in acquisition of recombinant plasmid termed Ad5_Standard/pGEM*^®^*-T. Based on Ad5_Standard/pGEM*^®^*-T, the molecular mass number of copies per ng was calculated. The recombinant plasmid Ad5_Standard/pGEM*^®^*-T was isolated and used to prepare a standard curve during Adenovirus DNA quantification experiments.

DNA isolation was performed using High Pure Viral Nucleic Acid Kit (Roche Diagnostics), according to the manufacturer’s instructions. The concentration and purity of isolated DNA were assessed with a NanoDrop spectrophotometer (Thermo Fisher Scientific, Waltham, MA, USA). The number of HAdV genomic DNA copies was determined with quantitative PCR (qPCR) with primers (5′-GGA CGC CTC GGA GTA CCT GAG-3′; 5′- ACA GTG GGG TTT CTG AAC TTG TT-3′), and probe (JOE-CTG GTG CAG TTC GCC CGT GCC-TAMRA) targeting viral hexon gene [23] in QuantStudio™ 5 Real-Time PCR System (Thermo Fisher Scientific, Waltham, MA, USA) with GoTaq*^®^* Probe qPCR Master Mix (Promega, Madison, WI, USA). A standard curve was prepared using serial 10-fold dilutions of Ad5_Standard/pGEM*^®^*-T (10^6^–10 copies per reaction). Data are presented as the HAdV DNA copy number per ng of the total isolated DNA.

### 2.10. Statistical Analysis

Statistically significant differences were determined with one-way analysis of variation (ANOVA) using the Student–Newman–Keuls multiple comparisons test in GraphPad Prism version 9 (GraphPad Software Inc., San Diego, CA, USA). Data were presented as mean ± standard deviation (SD) from at least three independent experiments. Statistical differences were interpreted as significant at *p* < 0.05 (*), highly significant at *p* < 0.01 (**), and not significant at *p* > 0.05 (ns).

## 3. Results

### 3.1. HAdVs Correlation with CAR during Entry into Neurons

**Figure 1:**
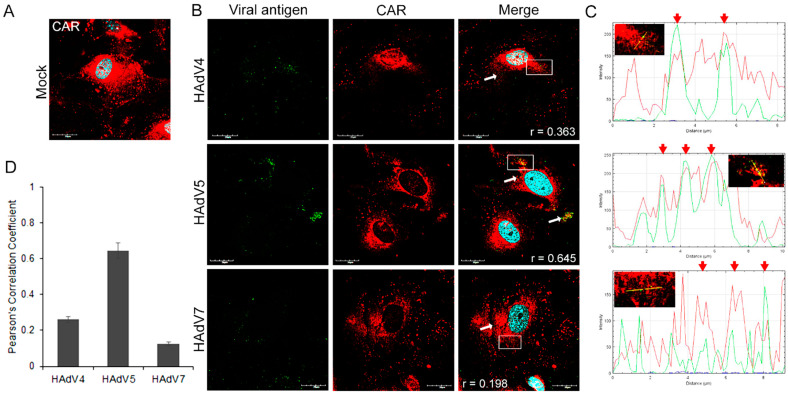
Localization of CAR in mock-infected (**A**) and HAdVs-infected (10 min pi) (**B**) primary murine neurons. White arrows mark the virus and CAR accumulation sites, whereas white squares indicate regions where the fluorescence intensity measurement was detected. (**C**) Immunofluorescence analysis of HAdV4, 5, 7, and CAR localization. The line profile plots indicate the intensity distribution of green and red channels through the yellow lines in the magnified view of ROI in the merged panel. Red arrows show the overlay of the red and green channels. (**D**) Quantification of correlation employing Fiji/ImageJ Coloc 2 plugin: the graph represents Pearson’s coefficient of HAdV antigens and CAR correlation from ten independent fields of cells in two experiments (data represent mean ± standard error of the mean). CAR—red; HAdV antigens—green; DNA—blue. Scale bars: 20 µm.

Since CAR was identified as a primary receptor for adenovirus cell binding, we examined its expression in the primary murine neurons (Figure 1A). Immunofluorescence analysis was performed to track the cellular distribution of CAR in HAdV-infected neurons. At 10 min post-infection (pi), positive signals for adenoviral antigen were located on the cell’s periphery, where positive signals for CAR were also observed. We did not observe differences in CAR distribution in HAdV4-, 5-, or 7-infected cells. Confocal image analysis revealed the overlap of viral antigen and receptor signals (Figure 1B, white arrows). The fluorescence intensity of two channels—red for CAR and green for HAdV antigens—was measured along the yellow line and presented as line profile plots. We observed an overlap of the red and green channels (Figure 1C, red arrows). Next, we used these images for correlation analyses by applying the Coloc2 measurement option of the image analysis software Fiji/ImageJ (NIH Image, version 1.53a, USA), which delivers an analysis of pixel-intensity correlation. The determination of Pearson’s coefficient (r), which indicates the correlation between HAdV antigens and CAR, was performed in ten independent fields of cells in two different experiments. According to the results obtained, we can observe that CAR strongly correlated with HAdV5 (r = 0.645). The Pearson correlation coefficient for HAdV4/CAR was 0.260, and it was 0.125 for HAdV7/CAR (Figure 1D), indicating a weak correlation. These results suggest that HAdVs correlate with CAR in murine neurons, and this correlation depends on the adenovirus serotype.

### 3.2. Localization of HAdV, Clathrin, and Caveolin during Internalization

**Figure 2:**
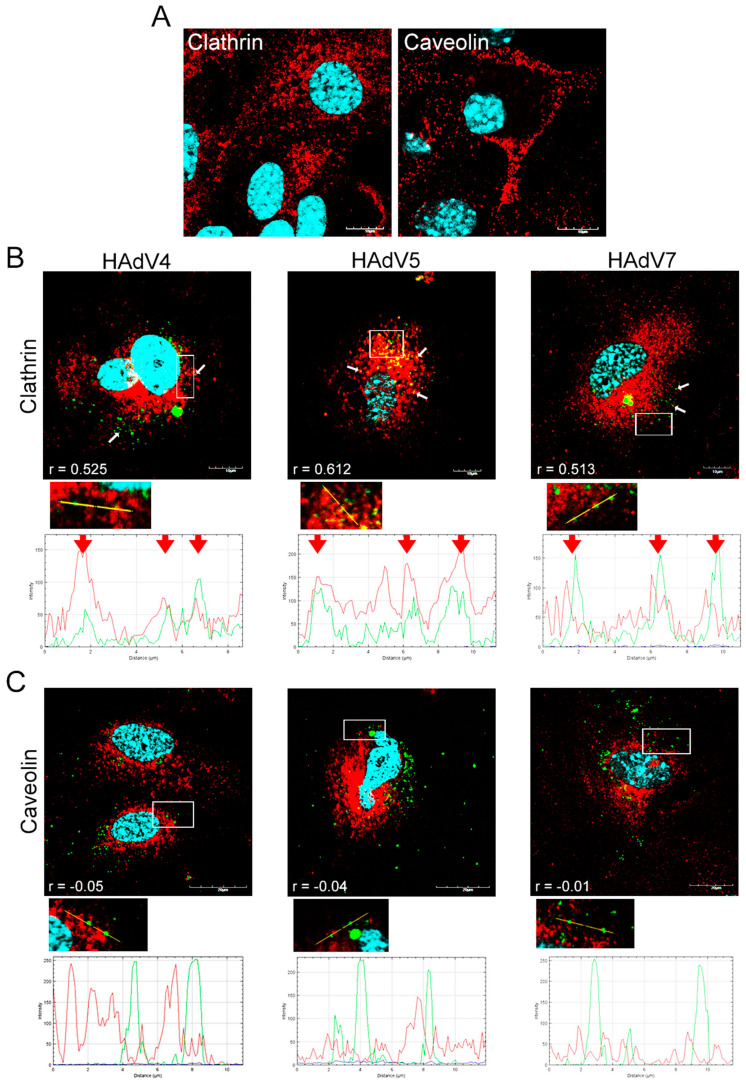
Immunofluorescence images of clathrin and caveolin-1 in mock-infected (**A**) and HAdVs-infected (15 min pi) (**B**) primary murine neurons. A large amount of virions accumulated at the site of clathrin occurrence after internalization (white arrows). White squares indicate regions where the detection of fluorescence intensity measurement was performed. The line profile plots (**B**,**C**) indicate the intensity distribution of green and red channels through the yellow lines in the magnified view of ROI in the merged panel. Red arrows show an overlay of the red and green channels (**B**). Clathrin/caveolin proteins—red; HAdV antigens—green; DNA—blue. Scale bars: 10 µm (**A**,**B**), 20 µm (**C**).

**Figure 3:**
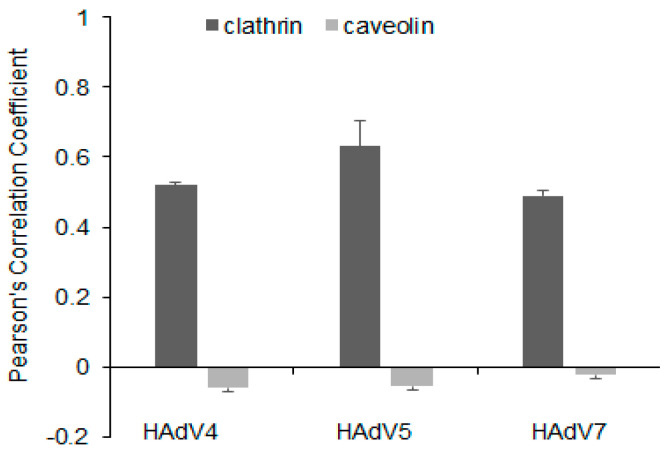
Quantification of correlation between clathrin/caveolin and HAdV4, 5, 7 in murine neurons. Pearson’s correlation coefficients (r) were calculated from ten independent fields of cells in two different experiments employing Fiji/ImageJ Coloc 2 plugin. Data representing mean r ± standard error of the mean are indicated on bar charts.

In the current study, clathrin and caveolin-1 proteins were labeled for clathrin- and caveolin-dependent endocytic vesicles in neurons, respectively (Figure 2A). Immunofluorescence images were used to analyze whether one of two classical endocytic pathways might play a role in HAdV internalization in neurons. Our results showed that at 15 min post-infection (pi), positive signals for viral antigen were mainly distributed near the cell membrane, and most of them were observed to overlap with positive signals for clathrin (Figure 2B). To confirm these observations, an analysis of pixel-intensity correlation using the Fiji/ImageJ Coloc 2 plugin was performed. HAdV 4, 5, and 7 correlated with clathrin proteins with Pearson’s correlation coefficient of 0.521, 0.630, and 0.498, respectively, indicating moderate correlation (Figure 3). On the other hand, double immunofluorescence staining and correlation analysis showed a weak negative correlation (Figure 2C and Figure 3). These findings demonstrated that the entry of tested HAdVs into neurons was most likely associated with clathrin but not caveolin protein.

### 3.3. Distribution of EEA1 and Rab5 Proteins in HAdV-Infected Neurons

**Figure 4:**
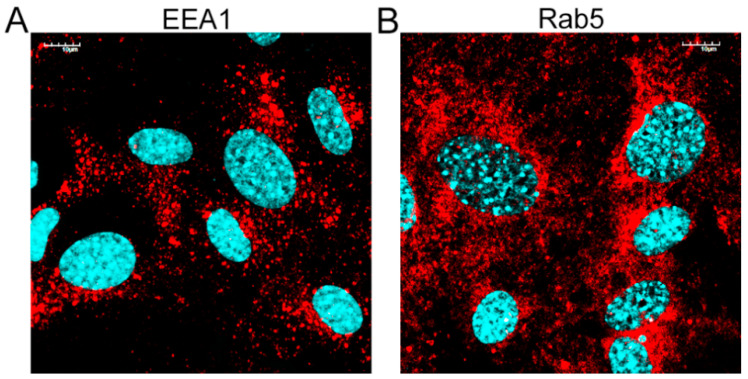
Localization of the early endosomal proteins in primary murine neurons. Immunofluorescence images of EEA1 (**A**) and Rab5 (**B**) proteins in mock-infected neurons. EE markers (EEA1/Rab5)—red; DNA—blue. Scale bars: 10 µm.

Next, we performed an immunofluorescence analysis of the cellular distribution of early endosomal proteins (EEA1/Rab5) in HAdV-infected murine neurons. Double immunofluorescence was performed to track the distribution of HAdV and early endosomes at 15, 30, 60, and 120 min pi. In mock-infected neurons, EEA1 and Rab5 proteins localized in the early endosomes were visible as punctate or dot-like structures (Figure 4).

**Figure 5:**
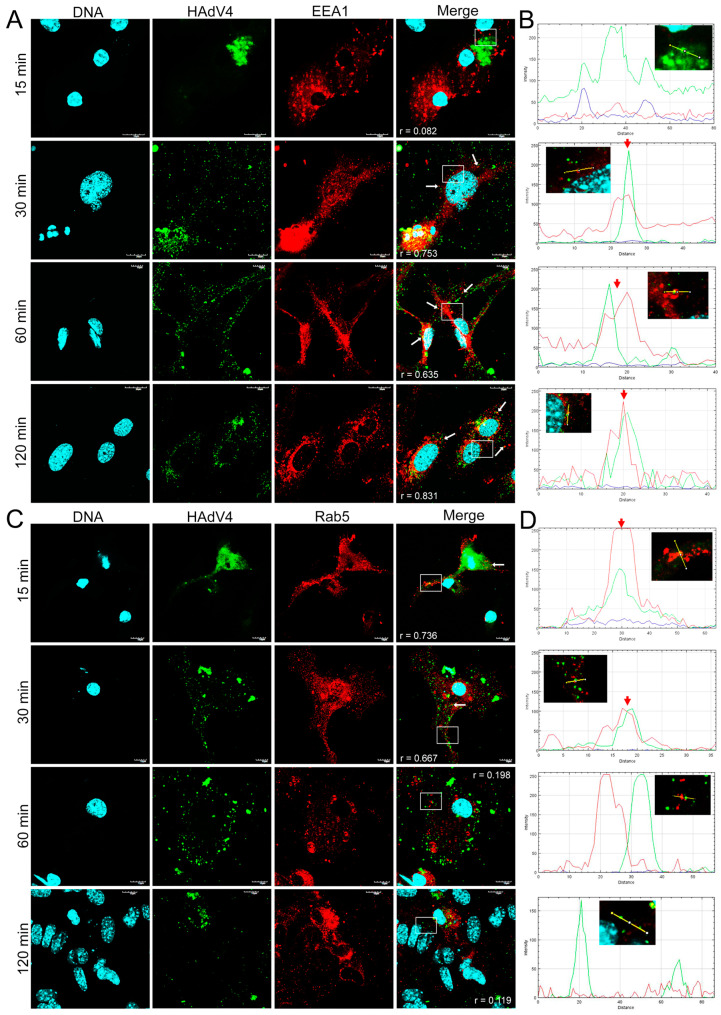
Immunofluorescence images of EEA1 (**A**) and Rab5 (**C**) proteins in HAdV4-infected neurons at 15, 30, 60, and 120 min pi. White arrows mark the site of virus and EE markers’ accumulation, whereas white squares indicate regions where fluorescence intensity measurement was detected. The line profile plots (**B**,**D**) indicate the intensity distribution of green and red channels through the yellow lines in the magnified view of ROI in the merged panel. Red arrows show an overlay of the red (Rab5/EEA1) and green (HAdV4) channels. EE markers (EEA1/Rab5)—red; HAdV antigens—green; DNA—blue. Scale bars: 10 µm and 20 µm.

**Figure 6:**
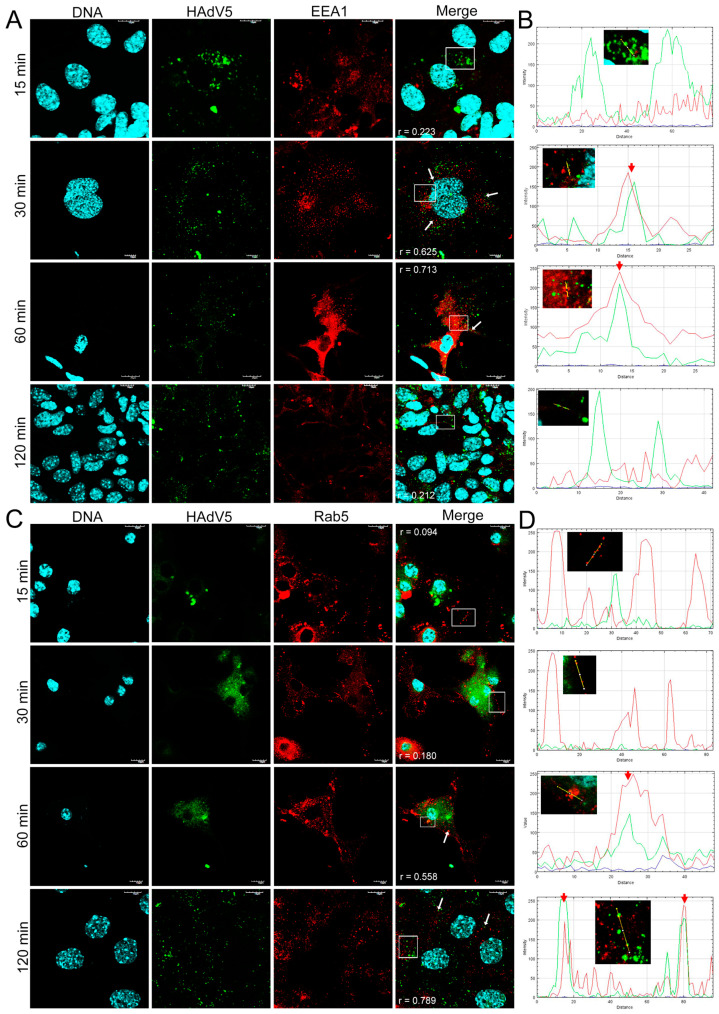
Immunofluorescence images of EEA1 (**A**) and Rab5 (**C**) proteins in HAdV5-infected neurons at 15, 30, 60, and 120 min pi. White arrows mark the site of virus and EE markers’ accumulation, whereas white squares indicate regions where fluorescence intensity measurement was detected. The line profile plots (**B**,**D**) indicate the intensity distribution of green and red channels through the yellow lines in the magnified view of ROI in the merged panel. Red arrows show an overlay of the red (Rab5/EEA1) and green (HAdV5) channels. EE markers (EEA1/Rab5)—red; HAdV antigens—green; DNA—blue. Scale bars: 10 µm and 20 µm.

**Figure 7:**
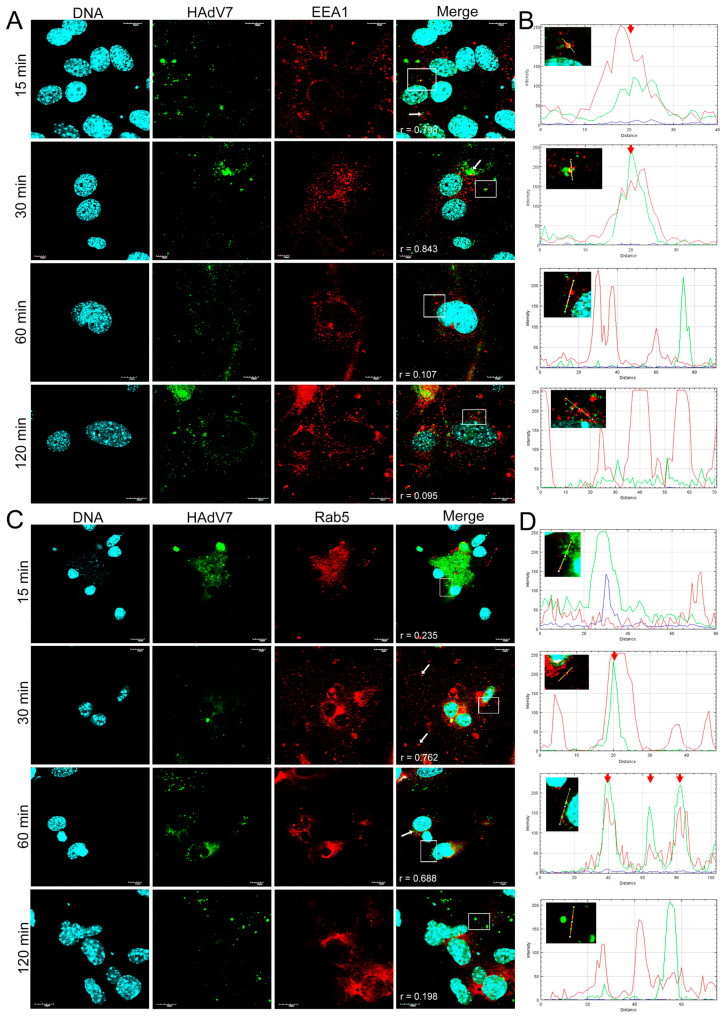
Immunofluorescence images of EEA1 (**A**) and Rab5 (**C**) proteins in HAdV7-infected neurons at 15, 30, 60, and 120 min pi. White arrows mark the site of virus and EE markers’ accumulation, whereas white squares indicate regions where fluorescence intensity measurement was detected. The line profile plots (**B**,**D**) indicate the intensity distribution of green and red channels through the yellow lines in the magnified view of ROI in the merged panel. Red arrows show an overlay of the red (Rab5/EEA1) and green (HAdV7) channels. EE markers (EEA1/Rab5)—red; HAdV antigens—green; DNA—blue. Scale bars: 10 µm and 20 µm.

**Figure 8:**
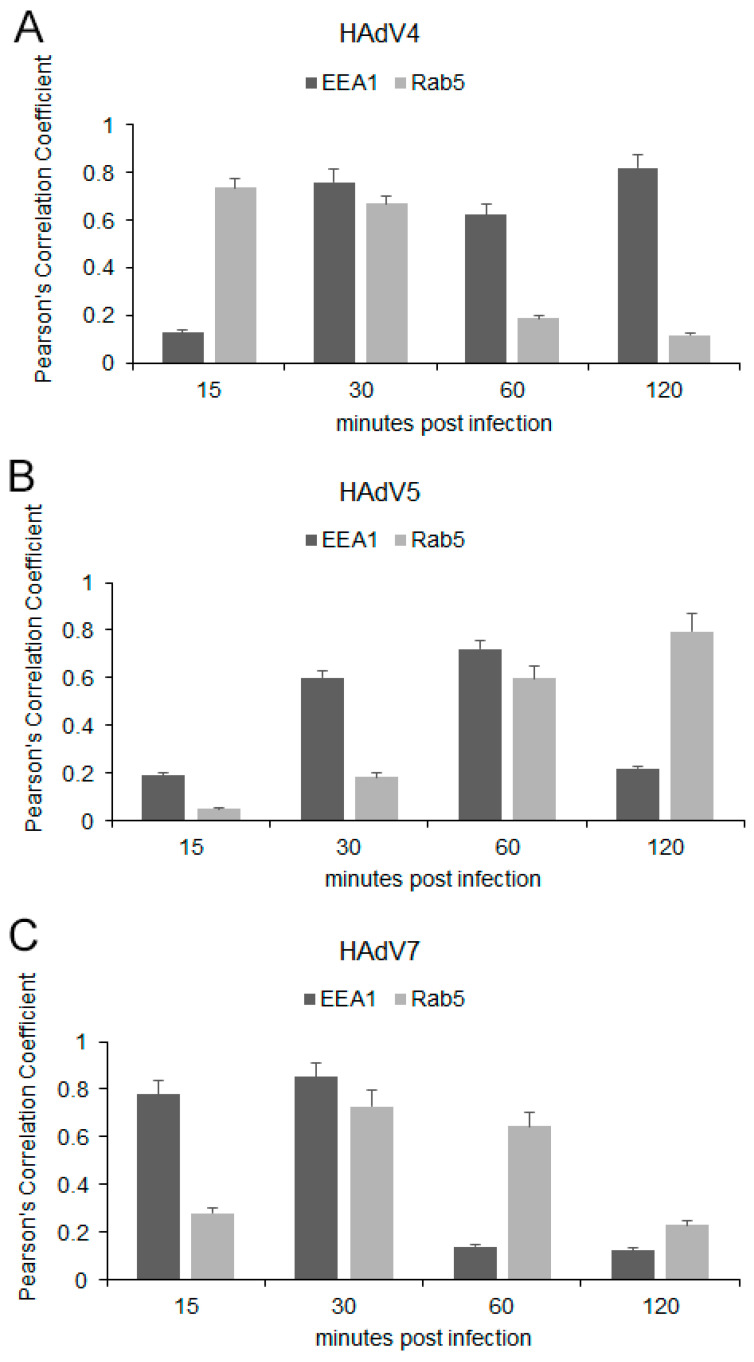
Quantification of correlation between EEA1/Rab5 proteins and HAdV4 (**A**), 5 (**B**), 7 (**C**) in murine neurons. Pearson’s correlation coefficients (r) were calculated from ten independent fields of cells in two different experiments employing the Fiji/ImageJ Coloc 2 plugin. Data representing mean r ± standard error of the mean are indicated on bar charts.

In a virus entry assay monitored by indirect immunofluorescence, the HAdV4 and 5 antigens were located in the place of accumulation of EEA1 proteins at 30 min pi (Figure 5A and Figure 6A). In addition, HAdV4 remained longest correlated with early endosomes, also at 1 and 2 hpi (Figure 5A). For HAdV5, the accumulation of viral antigen with the EEA1 protein was not detected at subsequent time points (after 1 hpi) (Figure 6A). In the case of HAdV7, the accumulation of the viral antigen in the site of EEA1 protein occurrence was already found at 15 min pi (Figure 7A). A correlation analysis performed with Fiji/ImageJ confirmed the data obtained via confocal microscopy. The graphs show the overlapping fluorescence of the viral antigen (green channel) with the EEA1 protein (red channel) (Figure 5A, Figure 6A and Figure 7A; red arrows). The calculation of Pearson’s correlation coefficients confirmed our observations, indicating a strong correlation between HAdV and EEA1 protein. HAdV4 correlated with EEA1 at 30 (r = 0.760), 60 (r = 0.623), and 120 (r = 0.820) min pi; HAdV5 correlated at 30 (r = 0.601) and 60 (r = 0.721) min pi, whereas HAdV7 correlated at 15 (r = 0.779) and 30 (r = 0.853) min pi (Figure 8).

Using the same approach, we also tracked the localization of HAdVs and Rab5 protein in neurons. It was found that HAdV4 accumulated in the site of early endosomes expressing Rab5 protein already at 15 min pi (Figure 5B). In turn, HAdV5 was visible in the early endosomes at 60 and 120 min pi (Figure 6B). In the case of HAdV7, such accumulation was observed at 30 and 60 min pi (Figure 7B). Regarding the EEA1 endosomal protein, the correlation of viral antigen with Rab5 endosomal protein was confirmed by the Fiji/ImageJ program analysis. An overlap of the red and green channels is presented as a line profile plot (Figure 5B, Figure 6B and Figure 7B; red arrows). The measurement of Pearson’s correlation coefficients revealed that HAdV4 correlated with Rab5 at 15 (r = 0.736) and 30 (r = 0.671) min pi; HAdV5 correlated at 60 (r = 0.599) and 120 (r = 0.796) min pi, whereas HAdV7 correlated at 30 (r = 0.728) and 60 (r = 0.646) min pi (Figure 8A–C). Therefore, immunofluorescence assays and determination of Pearson’s correlation coefficient showed that HAdV correlated with markers of early endosomes markers during entry into neurons.

### 3.4. Effect of Endocytosis Inhibition on HAdV Infection

**Figure 9:**
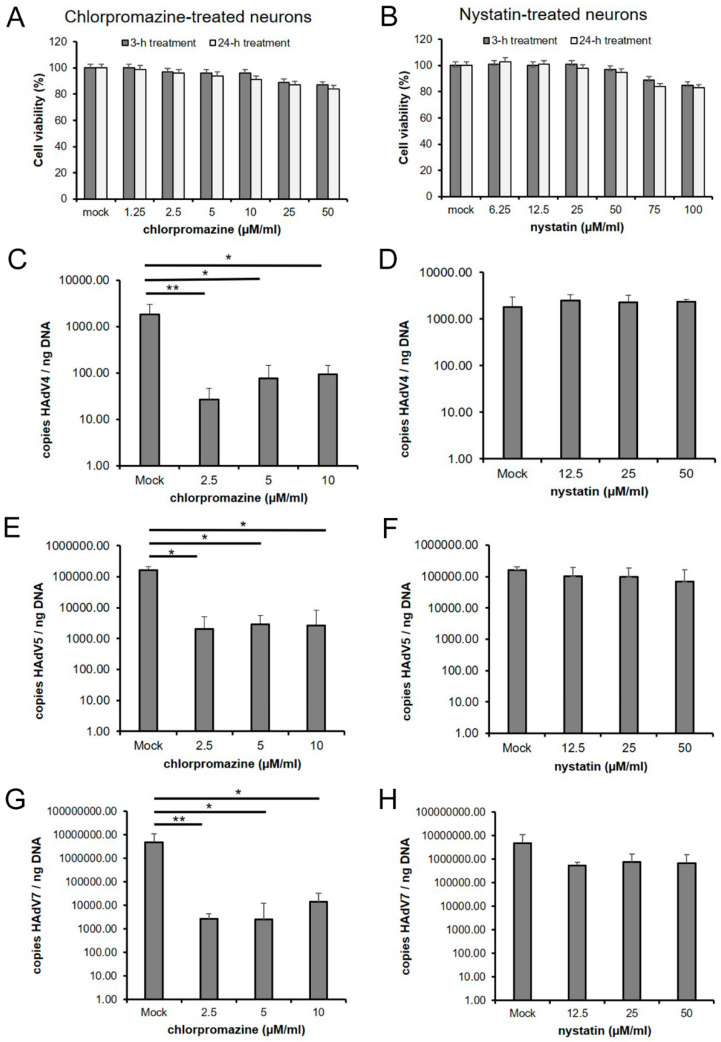
Inhibition of HAdV infection in primary murine neurons via chemical blocking of clathrin- and caveolin-mediated endocytosis. The effect of chlorpromazine and nystatin treatment on cell viability was measured by XTT assay (3 and 24 h treatment) (**A**,**B**). Cell viability was calculated as a percentage of viable, non-treated cells; columns represent the mean viability ± standard deviation (SD) (n = 3) after exposure to the inhibitor. Comparison of the viral DNA (copies/ng DNA) yield in mock-treated cells infected with HAdV 4, 5, or 7 and infected neurons treated with increasing concentrations of chlorpromazine (**C**,**E**,**G**) and nystatin (**D**,**F**,**H**) was performed by quantitative PCR (qPCR). Results are presented as mean ± SD of three experiments. Statistical comparisons were made between mock-treated HAdV-infected cells and HAdV-infected neurons treated with inhibitors (* *p* < 0.05; ** *p* < 0.01).

In order to study whether clathrin- and/or caveolin-mediated endocytosis were involved in HAdV entry, chlorpromazine and nystatin were used in these experiments. To study the toxicity of chlorpromazine and nystatin, primary murine neurons were cultured in the presence of different concentrations of each inhibitor for 3 and 24 h, and cell viability was assessed by XTT assay (Figure 9A,B). For further experiments, the highest non-toxic concentrations of chlorpromazine (2.5, 5, 10 µM) and nystatin (12.5, 25, 50 µM) were used.

Primary neurons were pre-treated for 1 h with the inhibitors and then infected with HAdV for 2 h. Quantitative real-time PCR was used to evaluate whether the tested inhibitors affected the uptake of the virus into neurons. Treatment of the neurons with chlorpromazine reduced all tested HAdV serotype infections; however, the most significant reduction in viral DNA copies was observed in HAdV7. Compared to the positive control, viral DNA copies were at 10^6^. In contrast, after chlorpromazine administration at the different concentrations, the values reached 10^3^ copies per ng DNA (Figure 9G). Administration of chlorpromazine to HAdV4- and HAdV5-infected neurons resulted in a two-logarithm decrease in viral DNA copies compared to the untreated control (HAdV4 from 10^3^ to 10^1^ and HAdV5 from 10^5^ to 10^3^) (Figure 9C,E). In turn, nystatin did not significantly affect viral uptake, indicating that caveolin may not play a role in viral internalization (Figure 9D,F,H). According to the obtained results, we can speculate that HAdV enters the primary murine neurons, most likely via the clathrin-dependent route.

## 4. Discussion

Human adenoviruses (HAdVs) are a significant cause of acute respiratory, ocular, and gastrointestinal diseases in humans. Although rare, adenoviral infections may present central nervous system dysfunction, causing meningitis and encephalitis, typically in the setting of pulmonary or disseminated disease. From brain tissue and cerebrospinal fluids (CSF), specific serotypes of adenoviruses—including type 1, 2, 3, 5, 7, 12, 26, and 32—have already been isolated [1,2,3,4,5,6]; however, the mechanism of adenoviral neuroinfection is still poorly understood. Therefore, in the current study, we investigated the early events during HAdV entry into neurons.

As mentioned above, HAdVs are capable of infecting nerve cells due to the presence of CAR receptor on the surface of their cell membrane [9]. CAR is a conserved receptor, and it is also found on cells of species other than humans, thanks to which it is possible to infect, e.g., mouse cells with human adenovirus serotypes [24]. In the present study, the primary culture of murine neurons was used as a model for studying the mechanism of neuronal uptake of adenoviruses. Although the replication of human adenoviruses is mainly restricted to human cells, reports indicate that some cell lines from other species support the complete replication cycle of adenovirus, although with reduced efficiency compared to human cells [25,26]. Our previous research has shown that human adenoviruses (HAdV4, 5, and 7) exhibit tropism to the neurons of Balb/c mice without the need for adaptation. We also provided evidence for the replication of all tested HAdVs serotypes in this model system [27].

In the current study, we focused on analyzing the distribution of signals in fluorescence confocal microscopy images to determine whether two molecules of interest correlate with each other. However, adopting this approach, a molecular interaction can indeed not be claimed. The relationship between two fluorescent signals can be estimated by calculating the correlation coefficient, known as Pearson’s correlation coefficient (r). It can range in value from −1 to 1, evaluating whether the results are positively correlated, negatively correlated, or uncorrelated. Immunofluorescence imaging techniques were used to determine the correlation of the viral antigen of various HAdV types with the CAR receptor, proteins participating in receptor-dependent endocytosis (clathrin, caveolin), and early endosome proteins (EEA1, Rab5). Confocal images were used for correlation analysis employing the Coloc 2 plugin of the extended ImageJ version Fiji [28,29,30].

Since the best studied adenovirus receptor is the coxsackievirus and adenovirus receptor (CAR) [31], we decided to examine its expression in murine neurons infected with HAdV serotypes. According to literature data, CAR occurs in cells of various organs, including the brain, and its expression allows the entry of adenoviruses belonging to subgroups A (HAdV12), C (HAdV2, HAdV5), E (HAdV4), and F (HAdV41) [32]. The correlation of adenoviral antigen and CAR, shown as Pearson’s coefficient, indicated that the HAdV4 and HAdV5 types correlated with CAR and that the correlation was higher for HAdV5 (r > 0.6). A moderate-to-weak correlation was demonstrated for HAdV4 (r = 0.26–0.5), and a weak-to-negligible correlation was established for HAdV7 (r = 0.1–0.25).

Studies on HAdVs using caveolin-mediated endocytosis for cell entry have not been as extensive as studies on clathrin-mediated endocytosis. Research conducted by Colin et al. (2005) revealed that infection of mature plasmocytic cells with HAdV5 was not the result of clathrin-dependent endocytosis but caveolae-mediated entry, as the colocalization of HAdV5 with caveolae/lipid rafts was detected [33]. Since the mechanism of neuronal uptake of HAdVs has not been investigated, we decided to find out which of these proteins could be involved in the entry of different adenovirus serotypes. Immunofluorescence analysis revealed the accumulation of viral particles where clathrin occurred and the lack of such accumulation with caveolin for all tested HAdV serotypes. The quantification of correlation between clathrin and HAdVs using Pearson’s coefficient indicated a moderate-to-strong correlation (r = 0.5–0.75). To confirm this observation, chlorpromazine, which prevents the assembly of clathrin-coated pits, was used to inhibit clathrin-mediated uptake of viruses, and nystatin, a cholesterol-sequestering drug, was employed to block caveola-mediated endocytosis. According to the results obtained by qPCR, virus entry was significantly lower in chlorpromazine-treated neurons than in mock-treated control cells. Thus, chlorpromazine significantly reduced HAdV uptake in neurons, whereas nystatin did not appear to affect viral entry. According to the obtained results, the admission of all tested HAdV types into neurons was most likely associated with clathrin but not caveolin.

Early endosomal proteins play crucial roles in regulating vesicle sorting, docking, and fusion. Rab5, as one of the essential small GTPases, is involved in the regulation of fusion between clathrin-coated vesicles and early endosomes [34]. This endosomal protein also regulates interactions with the microtubule system; it stimulates the association of early endosomes with microtubules and early endosome movement toward the minus ends of microtubules [35]. The Rab5 effector early endosome antigen 1 (EEA1) constitutes a sub-domain antigen for early endosomes, coordinating their sorting and docking in cells. EEA1 proteins are present in the neuron’s dendrites due to the presence of the MAP2 marker [36].

Based on the analysis of confocal microscopy images, it was found that all tested HAdV serotypes accumulated with early endosomes when penetrating murine neurons in vitro; however, this was observed at different times post-infection, depending on the HAdV serotype used. The obtained results indicate that HAdV serotype 7 accumulated with the EEA1 protein already at 15 min. A similar colocalization of HAdV2 with the EEA1 protein was observed in HeLa cells (human epithelial carcinoma cell line). At 15 min after infection, 7.5% of all wild-type HAdV2 particles introduced into the cell were found to be colocalized with the EEA1 protein [37]. For HAdV5, the accumulation of viral antigen with the EEA1 protein was observed at 30 min pi, but it was not detected at subsequent time points. The same analysis was performed for the Rab5 protein, which is crucial in transporting HAdVs into the cell [13]. As with the EEA1 protein, we showed that the HAdVs/Rab5 correlation depends on the virus serotype. Correlations of HAdV4 with the Rab5 protein appeared in the initial penetration stage at 15 and 30 min pi, while HAdV5 correlated with this protein later, at 60 and 120 min pi. On the other hand, for HAdV7, the correlation was confirmed at 30 and 60 min pi. The quantification of r values within the range of 0.6–0.9 demonstrated a moderate-to-strong correlation of HAdVs and EEA1/Rab5 at the indicated time points after infection.

In conclusion, the data presented in this study showed that HAdV correlates with the coxsackievirus and adenovirus receptor (CAR), clathrin, and early endosomal proteins (EEA1, Rab5) in primary murine neurons. To our knowledge, this is the first study analyzing the neuronal uptake and early events of HAdV, the replication of which has been confirmed in primary murine neurons [23]. Based on the results presented, we can assume that HAdVs use the CAR receptor and clathrin-dependent endocytosis when entering neurons. However, further research will be required to verify this hypothesis. Nevertheless, we showed that HAdV strongly correlates with the CAR receptor and Rab proteins on early vesicles, which provides helpful clues for explaining the early events of adenoviruses during neuron infection. Understanding the mechanisms of HAdV entry into neurons is essential not only because of a better understanding of the mechanism of adenoviral neuroinfections but also because HAdVs are the most deployed viral platform for cancer applications. Recombinant adenoviral vectors have been widely studied for their use in targeting genes in the nervous system [38]. Additionally, the tropism of the above serotypes for neurons and the well-characterized mechanism of intracellular entry and intracellular transport may enable their use as vectors in the treatment of diseases such as brain tumors or neurodegenerative disorders.

## Data Availability

Data available from authors at request.

## References

[1] Lynch J.P., Kajon A.E. (2016). Adenovirus: Epidemiology, Global Spread of Novel Serotypes, and Advances in Treatment and Prevention. Semin. Respir. Crit. Care Med..

[2] Lion T. (2014). Adenovirus infections in immunocompetent and immunocompromised patients. Clin. Microbiol. Rev..

[3] Huang Y.C., Huang S.L., Chen S.P., Huang Y.L., Huang C.G., Tsao K.C., Lin T.Y. (2013). Adenovirus infection associated with central nervous system dysfunction in children. J. Clin. Virol..

[4] Dubberke E.R., Tu B., Rivet D.J., Storch G.A., Apisarnthanarak A., Schmidt R.E., Weiss S., Polish L.B. (2006). Acute meningoencephalitis caused by adenovirus serotype 26. J. Neurovirol..

[5] Frange P., Peffault de Latour R., Arnaud C., Boddaert N., Oualha M., Avettand-Fenoel V., Bernaudin F., Aguilar C., Barnerias C., Leruez-Ville M. (2011). Adenoviral infection presenting as an isolated central nervous system disease without detectable viremia in two children after stem cell transplantation. J. Clin. Microbiol..

[6] Chatterjee N.K., Samsonoff W.A., Balasubramaniam N., Rush-Wilson K., Spargo W., Church T.M. (2000). Isolation and characterization of adenovirus 5 from the brain of an infant with fatal cerebral edema. Clin. Infect. Dis..

[7] Nestić D., Božinović K., Pehar I., Wallace R., Parker A.L., Majhen D. (2021). The Revolving Door of Adenovirus Cell Entry: Not All Pathways Are Equal. Pharmaceutics.

[8] Słońska A., Cymerys J., Bańbura M.W. (2016). Mechanisms of endocytosis utilized by viruses during infection. Post. High. Med. Dośw..

[9] Wiethoff C.M., Nemerow G.R. (2015). Adenovirus membrane penetration: Tickling the tail of a sleeping dragon. Virology.

[10] Meier O., Greber U.F. (2004). Adenovirus endocytosis. J. Gene. Med..

[11] Dorner A.A., Wegmann F., Butz S., Wolburg-Buchholz K., Wolburg H., Mack A., Nasdala I., August B., Westermann J., Rathjen F.G. (2005). Coxsackievirus-adenovirus receptor (CAR) is essential for early embryonic cardiac development. J. Cell Sci..

[12] Amstutz B., Gastaldelli M., Kalin S., Imelli N., Boucke K., Wandeler E., Mercer J., Hemmi S., Greber U.F. (2008). Subversion of CtBP1-controlled macropinocytosis by human adenovirus serotype 3. EMBO J..

[13] Rauma T., Tuukkanen J., Bergelson J.M., Denning G., Hautala T. (1999). Rab5 GTPase regulates adenovirus endocytosis. J. Virol..

[14] Nemerow G.R. (2000). Cell receptors involved in adenovirus entry. Virology.

[15] Smith A.E., Helenius A. (2004). How viruses enter animal cells. Science.

[16] Wickham T.J., Mathias P., Cheresh D.A., Nemerow G.R. (1993). Integrins alpha v beta 3 and alpha v beta 5 promote adenovirus internalization but not virus attachment. Cell.

[17] Sirena D., Lilienfeld B., Eisenhut M., Kälin S., Boucke K., Beerli R.R., Vogt L., Ruedl C., Bachmann M.F., Greber U.F. (2004). The human membrane cofactor CD46 is a receptor for species B adenovirus serotype 3. J. Virol..

[18] Bucci C., Parton R.G., Mather I.H., Stunnenberg H., Simons K., Hoflack B., Zerial M. (1992). The small GTPase rab5 functions as a regulatory factor in the early endocytic pathway. Cell.

[19] McBride H.M., Rybin V., Murphy C., Giner A., Teasdale R., Zerial M. (1999). Oligomeric complexes link Rab5 effectors with NSF and drive membrane fusion via interactions between EEA1 and syntaxin 13. Cell.

[20] Cymerys J., Chodkowski M., Słońska A., Krzyżowska M., Bańbura M.W. (2019). Disturbances of mitochondrial dynamics in cultured neurons infected with human herpesvirus type 1 and type 2. J. Neurovirol..

[21] Akoglu H. (2018). User’s guide to correlation coefficients. Turk. J. Emerg. Med..

[22] Schober P., Boer C., Schwarte L.A. (2018). Correlation Coefficients: Appropriate Use and Interpretation. Anesth. Analg..

[23] Rola A., Przybylski M., Dzieciatkowski T., Turowska A., Łuczak M. (2007). Detection of human adenoviruses with real-time PCR assay using TaqMan fluorescent probes. Med. Dosw. Mikrobiol..

[24] Wehbi A., Kremer E.J., Dopeso-Reyes I.G. (2020). Location of the Cell Adhesion Molecule “Coxsackievirus and Adenovirus Receptor” in the Adult Mouse Brain. Front. Neuroanat..

[25] Ganly I., Mautner V., Balmain A. (2000). Productive replication of human adenoviruses in mouse epidermal cells. J. Virol..

[26] Hallden G., Hill R., Wang Y., Anand A., Liu T.C., Lemoine N.R., Francis J., Hawkins L., Kirn D. (2003). Novel immunocompetent murine tumor models for the assessment of replication-competent oncolytic adenovirus efficacy. Mol. Ther..

[27] Cymerys J., Słońska A., Chodkowski M., Przybylski M., Bańbura M.W. (2016). Primary murine neurons as in vitro model for studying neuroinfections caused by human adenoviruses. Acta Virol..

[28] Bolte S., Cordelières F.P. (2006). A guided tour into subcellular colocalization analysis in light microscopy. J. Microsc..

[29] Dunn K.W., Kamocka M.M., McDonald J.H. (2011). A practical guide to evaluating colocalization in biological microscopy. Am. J. Physiol. Cell Physiol..

[30] Moser B., Hochreiter B., Herbst R., Schmid J.A. (2017). Fluorescence colocalization microscopy analysis can be improved by combining object-recognition with pixel-intensity-correlation. Biotechnol. J..

[31] Bergelson J.M., Cunningham J.A., Droguett G., Kurt-Jones E.A., Krithivas A., Hong J.S., Horwitz M.S., Crowell R.L., Finberg R.W. (1997). Isolation of a common receptor for Coxsackie B viruses and adenoviruses 2 and 5. Science.

[32] Zhang Y., Bergelson J.M. (2005). Adenovirus receptors. J. Virol..

[33] Colin M., Mailly L., Rogée S., D’Halluin J.C. (2005). Efficient species C HAdV infectivity in plasmocytic cell lines using a clathrin independent lipid raft/caveola endocytic route. Mol. Ther. J. Am. Soc. Gene Ther..

[34] Mc Lauchlan H., Newell J., Morrice N., Osborne A., West M., Smythe E. (1998). A novel role for Rab5-GDI in ligand sequestration into clathrin-coated pits. Curr. Biol..

[35] Nielsen E., Severin F., Backer J.M., Hyman A.A., Zerial M. (1999). Rab5 regulates motility of early endosomes on microtubules. Nat. Cell Biol..

[36] Wilson J.M., Hoop M., Zorzi N., Toh B.H., Dotti C.G., Parton R.G. (2000). EEA1, a Tethering Protein of the Early Sorting Endosome, Shows a Polarized Distribution in Hippocampal Neurons, Epithelial Cells, and Fibroblasts. Mol. Biol. Cell.

[37] Gastaldelli M., Imelli N., Boucke K., Amstutz B., Meier O., Greber U.F. (2008). Infectious adenovirus type 2 transport through early but not late endosomes. Traffic.

[38] Nandi S., Lesniak M.S. (2009). Adenoviral virotherapy for malignant brain tumors. Expert. Opin. Biol. Ther..

